# Isolation and Co-culture of Paneth Cells and Intestinal Stem Cells

**DOI:** 10.21769/BioProtoc.5449

**Published:** 2025-09-20

**Authors:** Ryosuke Isotani, Masaki Igarashi, Masaomi Miura, Toshimasa Yamauchi

**Affiliations:** Department of Diabetes and Metabolic Diseases, Graduate School of Medicine, The University of Tokyo, Tokyo, Japan

**Keywords:** Co-culture, Paneth cells, Intestinal stem cells, Crypt, Organoid

## Abstract

Crypts at the base of intestinal villi contain intestinal stem cells (ISCs) and Paneth cells, the latter of which work as niche cells for ISCs. When isolated and cultured in the presence of specific growth factors, crypts give rise to self-renewing 3D structures called organoids that are highly similar to the crypt-villus structure of the small intestine. However, the organoid culture from whole crypts does not allow investigators to determine the contribution of their individual components, namely ISCs and Paneth cells, to organoid formation efficiency. Here, we describe the method to isolate Paneth cells and ISCs by flow cytometry and co-culture them to form organoids. This approach allows the determination of the contribution of Paneth cells or ISCs to organoid formation and provides a novel tool to analyze the function of Paneth cells, the main component of the intestinal stem cell niche.

Key features

• This protocol introduces the method for isolating Paneth cells and *Lgr5+* ISCs by flow cytometry and co-culturing them.

• This protocol allows analyzing the effect of genetic or biochemical modifications of Paneth or *Lgr5^+^* ISCs on organoid formation.

• This protocol provides a new platform to analyze Paneth cell function.

## Background

The homeostasis of each organ and tissue is maintained by its respective tissue stem cells. Excessive proliferation of stem cells can lead to carcinogenesis, while a decline in stem cell function results in impaired tissue function. Therefore, properly regulating the proliferation and differentiation functions of stem cells is crucial for disease prevention.

The homeostasis of the intestinal epithelium is maintained by the self-renewal and differentiation of the intestinal stem cells (ISCs) [1]. Lgr5^+^ cells comprise the majority of ISCs [2]. Crypts at the villi base contain ISCs and Paneth cells, which neighbor the Lgr5^+^ ISCs. While Paneth cells secrete antibacterial compounds such as lysozyme and defensins and control the intestinal flora, they are also an important source of niche signals for ISC maintenance, such as Wnt3 [3]. The Paneth cells can also respond to caloric restriction (CR) by downregulating the mammalian target of rapamycin complex 1 (mTORC1) to induce cyclic ADP ribose (cADPR) secretion [4], which mediates ISC proliferation [5]. When isolated ex vivo and cultured in the presence of specific growth factors and a Matrigel matrix, whole crypts produce self-renewing 3D structures called organoids that are highly similar to the structure of the adult small intestine [6]. However, organoid culture from whole crypts does not allow investigators to determine the contribution of Paneth cells to organoid formation. Here, we describe the method to isolate Paneth cells and Lgr5^+^ ISCs from crypts and co-culture them to form organoids. This method allows investigators to analyze the supportive function of Paneth cells subjected to genetic and biochemical modifications. Moreover, it is expected that this co-culture system could be applied in in vitro studies of drug discovery targeting Paneth cells.

## Materials and reagents

**Biological materials**

1. *Lgr5-EGFP-IRES-creERT2* mouse (The Jackson Laboratory, catalog number: 008875)

*Note: Mice to be used for this protocol need to be approved by the Institutional Animal Care and Use Committee of the institution.*

2. Cultrex R-Spondin 1 (RSPO1) cells (R&D Systems, catalog number: 3710-001-01)

**Reagents**

1. Phosphate-buffered saline (PBS) (FUJIFILM WAKO, catalog number: 045-29795)

2. Growth factor reduced Matrigel (Corning, catalog number: 356231)

3. Bovine serum albumin (BSA) (FUJIFILM WAKO, catalog number: 034-25462)

4. 0.5 M Ethylenediaminetetraacetic acid (EDTA) pH 8.0 (Nippon GENE Co., LTD, catalog number: 311-90075)

5. N-2 Supplement (100×) (Thermo Scientific, catalog number: 17502048)

6. B-27 supplement (50×), serum free (Thermo Scientific, catalog number: 17504044)

7. Y-27632 (FUJIFILM WAKO, catalog number: 259-00613)

8. Advanced DMEM F/12 (Thermo Scientific, catalog number: 12634010)

9. TrypLE Express (Thermo Scientific, catalog number: 12604-013)

10. DNAse1 recombinant RNase free (Sigma-Aldrich, catalog number: 0471672800)

11. MEMα (FUJIFILM WAKO, catalog number: 135-15175)

12. Penicillin-Streptomycin (FUJIFILM WAKO, catalog number: 168-23191)

13. N-Acetylcysteine (Sigma-Aldrich, catalog number: A9165)

14. HEPES solution (Sigma-Aldrich, catalog number: H0887)

15. GlutaMAX (Thermo Scientific, catalog number: 35050061)

16. Recombinant murine EGF (PeproTech, catalog number: 315-09)

17. Recombinant murine Noggin (PeproTech, catalog number: 250-38)

18. Afamin/Wnt3a CM (conditioned medium) (MBL, catalog number: J2-001)

19. Pacific blue anti-mouse CD24 (M1/69) antibody (BioLegend, catalog number: BL101819)

20. APC anti-mouse CD31 antibody (BioLegend, catalog number: 102509)

21. APC anti-mouse CD45 antibody (BioLegend, catalog number: 103111)

22. Propidium iodide (PI) (FUJIFILM WAKO, catalog number: 169-26281)

23. JAG-1 (AnaSpec, catalog number: AS-61298)

**Solutions**

1. Basal medium (see Recipes)

2. Organoid growth medium (see Recipes)

3. 1% BSA-PBS (see Recipes)

4. 5 mM EDTA-PBS (see Recipes)

5. Y-27632 stock solution (10 mM) (see Recipes)

6. N-Acetylcysteine stock solution (500 mM) (see Recipes)

7. Recombinant murine EGF stock solution (500 μg/mL) (see Recipes)

8. Recombinant murine Noggin stock solution (100 μg/mL) (see Recipes)

9. R-spondin 1 conditioned medium (see Recipes)

10. JAG-1 stock solution (1 mM) (see Recipes)

**Recipes**

**1. Basal medium**

**Table:** 

Reagent	Final concentration	Quantity or volume	Temperature	Shelf-life
Advanced DMEM F/12	n/a	10 mL	2–8 °C	12 months
GlutaMAX	2 mM	100 mL	15–30 °C	24 months
HEPES solution	10 mM	100 mL	2–8 °C	24 months
Penicillin-Streptomycin	1/100	100 mL	-20 °C	24 months
N-2 Supplement (100×)	1/100	100 mL	-5 to -20 °C	18 months
B-27 Supplement (50×)	1/50	200 mL	-5 to -20 °C	12 months
Y-27632 stock solution (10 mM)	10 mM	10 mL	-20 °C	24 months
Total	n/a	10 mL		

Prepare fresh.

**2. Organoid growth medium**

**Table:** 

Reagent	Final concentration	Quantity or volume	Temperature	Shelf-life
Basal media		5 mL		
N-Acetylcysteine stock solution (500 mM)	1 mM	10 mL	-2 to 8 °C	3 years
Recombinant murine EGF stock solution (500 mg/mL)	50 ng/mL	0.5 mL	-20 to -80 °C	12 months
Recombinant murine Noggin stock solution (100 mg/mL)	200 ng/mL	10 mL	-20 to -80 °C	12 months
R-spondin1 conditioning medium	1/30	200 mL	-20 to -80 °C	12 months
Afamin/Wnt3a CM	3/100	150 mL	-20 °C	24 months
Total	n/a	5 mL		

Prepare fresh.

**3. 1% BSA-PBS**

**Table:** 

Reagent	Final concentration	Quantity or volume	Temperature	Shelf-life
PBS		10 mL	15–30 °C	48 months
BSA	1%	100 mg	4 °C	48 months
Total	n/a	10 mL		

Filter sterilize 1% BSA-PBS through a syringe filter. Prepare fresh.

**4. 5 mM EDTA-PBS**

**Table:** 

Reagent	Final concentration	Quantity or volume	Temperature	Shelf-life
PBS		30 mL	15–30 °C	48 months
0.5 M EDTA solution	5 mM	300 mL	15–30 °C	3 years
Total	n/a	30 mL		

Prepare fresh.

**5. Y-27632 stock solution (10 mM)**

Y-27632 is dissolved in PBS at 10 mM and stored as a concentrated stock solution at -20 °C (shelf-life: 24 months).

**6. N-Acetylcysteine stock solution (500 mM)**

N-Acetylcysteine is dissolved in PBS at 500 mM and stored as a concentrated stock solution at -20 °C (shelf-life: 24 months).

**7. Recombinant murine EGF stock solution (500 mg/mL)**

Recombinant murine EGF is reconstituted with 0.1% BSA-PBS at 500 mg/mL and stored as a concentrated stock solution at -80 °C (shelf-life: 12 months).

**8. Recombinant murine Noggin stock solution (100 mg/mL)**

Recombinant murine Noggin is reconstituted with 0.1% BSA-PBS at 100 mg/mL and stored as a concentrated stock solution at -80 °C (shelf-life: 12 months).

**9. R-spondin 1 conditioned medium**

R-spondin 1 conditioned medium is produced according to the manufacturer’s manual (shelf-life: 12 months).

**10. JAG-1 stock solution (1 mM)**

JAG-1 is dissolved into PBS at 1 mM and stored as a concentrated stock solution at -80 °C (shelf-life: 6 months).

**Laboratory supplies**

1. 48-well tissue culture plate (IWAKI, catalog number: 3830-048)

2. 50 and 15 mL conical centrifuge tubes (Thermo scientific, catalog numbers: 339652 and 339650)

3. 10 mL serological pipette (Nippon Genetics, catalog number: FG3008)

4. 2 mL serological pipette (AS ONE Corporation, catalog number: 2-5237-12)

5. 1.5 mL tube (AS ONE Corporation, catalog number: 1-7521-01)

6. 10 mL syringe (TERUMO, catalog number: SS-10SZ)

7. Syringe filter (Sartorius, catalog number: S7597FX0SK)

8. 70 μM cell strainer (Corning, Falcon^®^, catalog number: 352350)

9. 40 μM cell strainer (Corning, Falcon^®^, catalog number: 352340)

10. p1000 pipette tips (Sorenson Bioscience, catalog number: 10130)

11. p200, p20 pipette tips (VIOLAMO, catalog number: V-200BN)

12. Hemocytometer (Thermo Fisher Scientific, Hirschmann^TM^, catalog number: 8100102)

13. FACS tube (Corning, Falcon^®^, catalog number: 352063)

14. Petri dish (Corning, Falcon^®^, catalog number:351029)

## Equipment

1. Pipet-Aid (Drummond Scientific Company, catalog number: 4-040-100J)

2. Micro-pipettes (GILSON: P1000, P200, P20)

3. Dissection forceps and scissors

4. Centrifuge (KUBOTA, model: 5922)

5. Brightfield inverted microscope (Nikon, model: ECLIPSE TS100)

6. Tube rotator at 4 °C (TAITEC, model: ROTATOR RT-50)

7. 37 °C, 5% CO_2_ cell culture incubator (Panasonic, model: MCO-19AICUV)

8. Biosafety cabinet (TOSC, model: BIO-LABO)

9. BD FACS Aria III Cell Sorter (BD Life Sciences)

## Procedure

**Figure 1.**
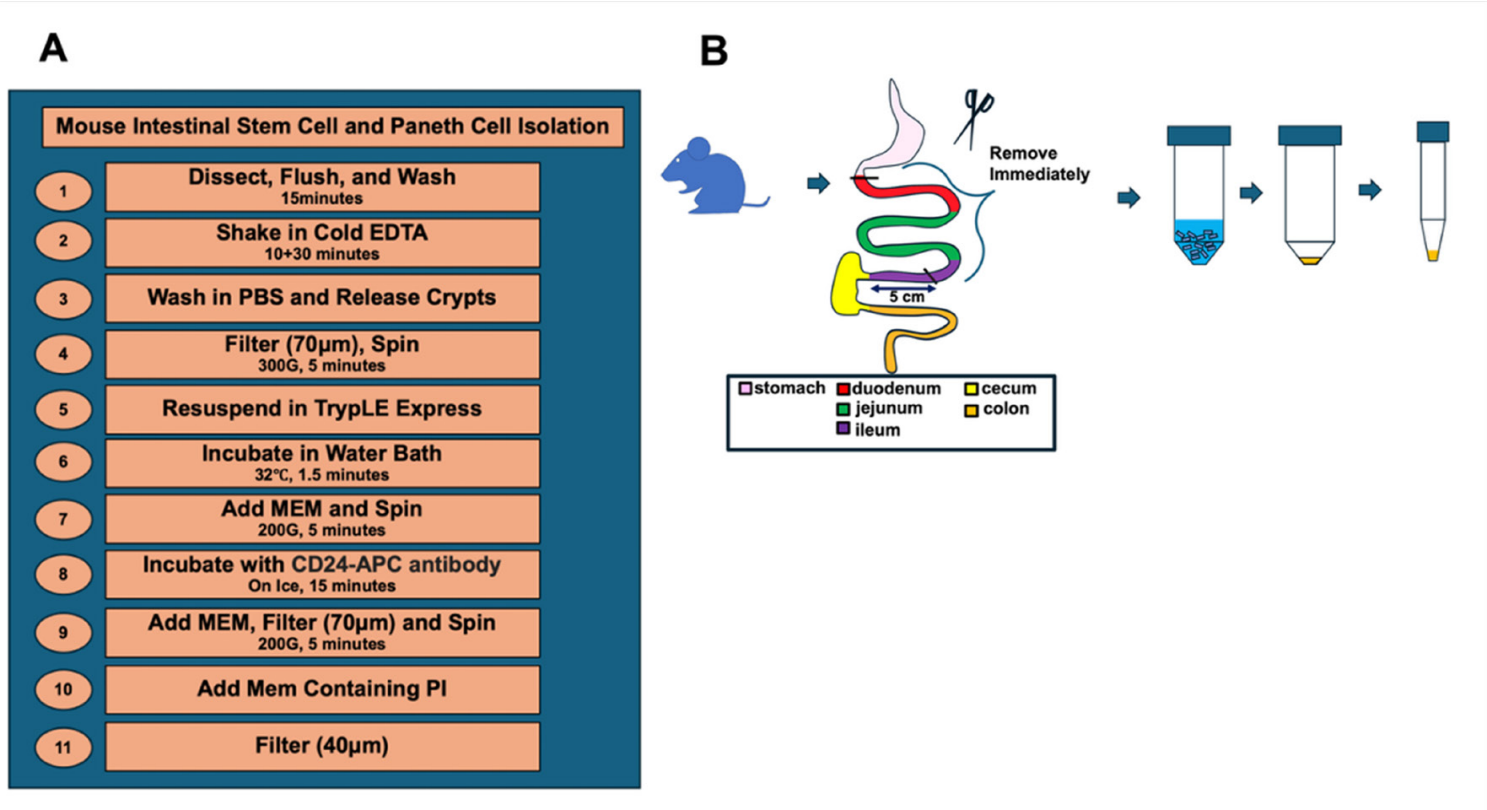
Overview and procedure scheme. An overview (A) and (B) schematic illustration of the key steps for the dissociation of intestinal crypt cells from murine small intestine.

**A. Isolation of crypts (Figure 1)**

1. Sacrifice *Lgr5-EGFP-IRES-creERT2* mouse in accordance with the approved animal use protocols at your laboratory’s sponsoring institution.

*Note: Per mouse, 20,000–50,000 ISCs and Paneth cells are obtained. How to sacrifice mice or their gender does not affect the co-culture performance. In older mice, the number of isolated ISCs decreases [7]. Throughout the whole protocol, a maximum of 4 animals can be processed at a time.*

2. Dissect and remove the whole small intestine, except 5cm of the ileal part, and promptly put it in ice-cold PBS in a Petri dish.

3. Use scissors to open the intestine longitudinally in ice-cold PBS and place it in a 50 mL conical tube containing 15 mL of ice-cold PBS. Invert 15 times to remove any visible debris.

4. Cut the intestine into 5 mm pieces and place them into 10 mL of ice-cold 5 mM EDTA-PBS. After shaking for 15 s, let the fragments settle by gravity for 30 s.

*Note: Steps A1–4 are shown in the attached video (Video 1).*

**Video 1.**
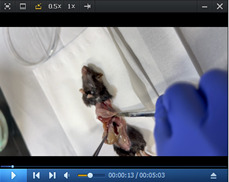
How to dissect a mouse, isolate the intestine, and cut into 5 mm pieces

5. Aspirate the supernatant, being careful to avoid the intestinal fragments, and replace with 10 mL of 5 mM EDTA-PBS. Place at 4 °C on a benchtop roller for 10 min (at the speed of 2.5 scale using the ROTATOR RT-50).

6. Repeat step A5 by aspirating the supernatant, replacing it with 10 mL of 5 mM EDTA-PBS, and then place it at 4 °C on a benchtop roller for 30 min.

7. Wait for fragments to settle by gravity on ice, aspirate the supernatant, gently add 10 mL of cold PBS to wash the crypts, and then vigorously shake for 15 s.

8. Collect this 10 mL supernatant fraction in a separate 15 mL tube (Fraction 1).

*Note: Fraction 1 is thrown away because it is a villus-rich fraction.*

9. Add 10 mL of cold PBS to the crypts again, vigorously shake for 15 s, and then collect this fraction in a separate 15 mL tube (Fraction 2).

10. Repeat step A9 three times to get a total of five fractions (Fractions 3–5).

11. Filter Fractions 2–5 through a 70 μm cell strainer into a 1% BSA-coated 50 mL tube and then spin the filtrate at 300× *g* for 5 min at 4 °C.

*Note: BSA coating is performed by shaking vigorously a 50 mL tube containing 1% PBS-BSA.*

12. Aspirate the supernatant.

*Note: The tissues and the crypts have to be kept on ice during the whole procedure of crypt isolation (steps A1–12).*

**B. Dissociation of crypt cells (Figure 1)**

1. Resuspend the pelleted crypts in 1.0 mL of TrypLE Express containing 100 μL of DNase I using a 2 mL pipette and move it into a 15 mL tube on ice.

*Note: Since TrypLE Express loses its effectiveness in ISC and Paneth cell isolation within 3–4 weeks after opening, it is recommended to prepare a fresh batch regularly.*

2. Immediately incubate the suspended crypts in a 37 °C water bath for 5 min and place on ice again.

*Note: Incubation for longer times or at higher temperatures could result in damage to the cells.*

3. Promptly move to a clean bench and add 12 mL of ice-cold MEMα. Gently mix by pipetting up and down twice using a 10 mL pipette and centrifuge at 200× *g* for 5 min at 4 °C. Aspirate the supernatant.

*Note: Steps B1–3 are particularly critical, as they can directly damage the cells. It is important to minimize any intervals between these steps as much as possible.*

4. Resuspend the pellets in 0.5 mL of ice-cold MEMα containing Pacific Blue–conjugated CD24 antibody, APC-conjugated CD31 antibody, and APC-conjugated CD45 antibody (1/500) and incubate on ice for 15 min. While waiting, prepare basal medium.

5. Add 12 mL of MEMα, triturate pellets, and filter through a 70 μm cell strainer into a 50 mL tube.

6. Transfer the content into a 15 mL tube and centrifuge at 200× *g* for 5 min at 4 °C. Aspirate the supernatant. While waiting, prepare basal media containing 1.5 μM PI.

7. Resuspend the pellets with 1.5 mL of basal medium containing 1.5 μM PI and transfer through a 40 μm cell strainer into a 50 mL tube.

8. Transfer the sample into a FACS tube.

*Note: The condition of the Pipet-Aid can influence isolated cell quality throughout section B. Frequent cleaning and replacement of the filter are especially important to minimize cellular damage.*

**C. Sorting of Paneth cells and ISCs**

1. Prepare two collection tubes (1.5 mL tubes) per sample containing 500 μL of basal medium.

*Note: Collection tubes are coated with 1% PBS-BSA.*

**Figure 2.**
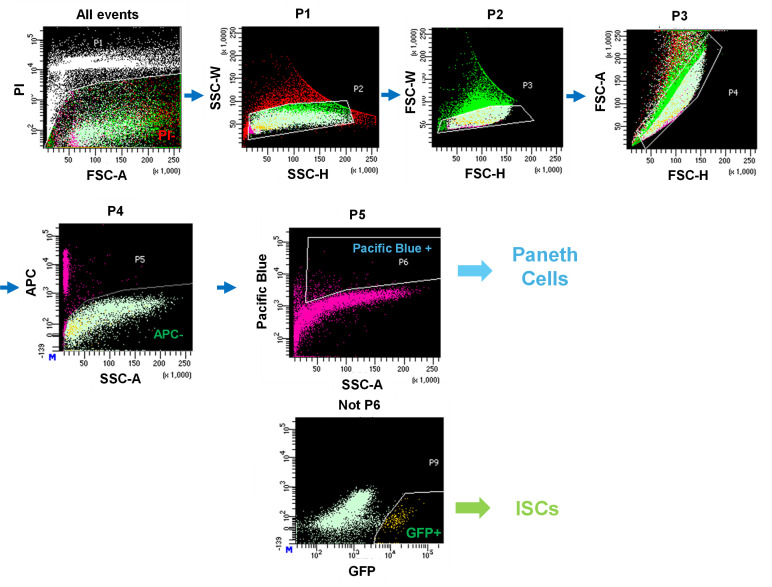
Gating strategy used for cell sorting by the BD FACS Aria III Cell Sorter. All events were first plotted based on FSC-A and PI to exclude dead cells (P1). Single cells were identified by SSC-H vs. SSC-W (P2) and FSC-H vs. FSC-W (P3). Subsequent gating on FSC-H vs. FSC-A (P4) excluded APC^+^ cells, yielding the APC- fraction (P5). Within this population, Paneth cells were identified as Pacific Blue^+^ cells (P6). The remaining Pacific Blue- fraction was further analyzed for GFP expression, and GFP^+^ cells were defined as Lgr5^+^ intestinal stem cells (ISCs, P9). Arrows indicate the sequential gating hierarchy.

2. Sort Paneth cells as CD24^hi^ SideScatter^hi^ Lgr5-EGFP^−^CD31^−^CD45^−^PI^−^ cells and ISCs as Lgr5-EGFP^hi^ CD24^−^ CD31^−^CD45^−^PI^−^ cells by FACS cell sorter. Gating for single-cell isolation is shown in Figure 2. Approximately 20,000–50,000 ISCs and Paneth cells per mouse are sorted into each collection tube.

*Note: Filter the samples through a 40-µm cell strainer again right before sorting, when you start sorting more than 1 h after preparation of dissociated cells.*

**D. Co-culture of Paneth cells and ISCs**

1. Centrifuge collection tubes at 300× *g* for 5 min at 4 °C.

2. Aspirate the supernatant. Total volume is adjusted to 30–100 μL depending on the number of sorted cells by basal medium.

3. Pick up 5 μL from a collection tube, put it on a hemocytometer, and count the number of cells under the microscope. Cell concentration should be 1,000–2,000 cells/µL.

4. Put 2,000 Paneth cells with 2,000 ISCs at the bottom of a new 1.5 mL tube on ice.

5. Prepare organoid growth medium.

6. Prepare 75% Matrigel diluted by organoid growth medium. Put JAG-1 (1/1,000: 1 μM) and Y-27632 (1/1,000: 10 μM) into diluted Matrigel.

*Notes:*

*1. The protein concentration of the original Matrigel should be 9–10 mg/mL.*

*2. JAG-1 in the Matrigel mixture stimulates Notch signaling, which is critical for ISC maintenance via interaction with Paneth cells.*

**Figure 3.**
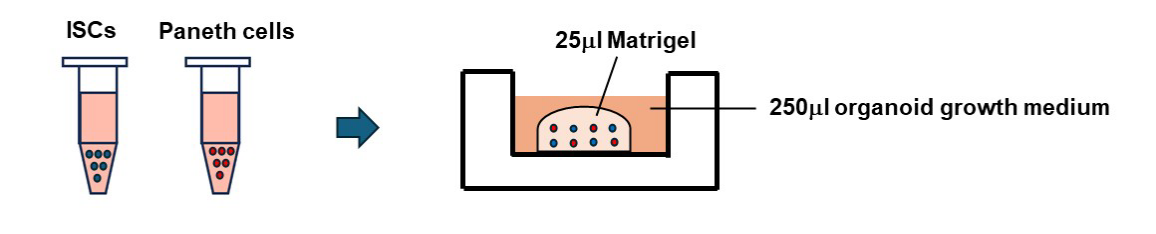
Schematic diagrams of the co-culture of Paneth cells and ISCs. Sorted cells, containing 2,000 stem cells and 2,000 Paneth cells, were embedded in 25 µL of Matrigel and plated in a dome shape. After 10 min, 250 µL of culture medium was gently overlaid.

7. Put 25 μL of prepared Matrigel on the mixture of ISCs and Paneth. Gently mix them and put the Matrigel drops with Paneth cells and ISCs on a 48-well plate (Figure 3).

8. Incubate the 48-well plate for 10 min in a 37 °C incubator to allow the Matrigel drops with ISCs and Paneth cells to solidify.

9. Put 250 μL of organoid growth media gently on the solidified Matrigel in each well.

*Note: Media does not have to be exchanged from day 1 to day 5 of culture.*

**Figure 4.**
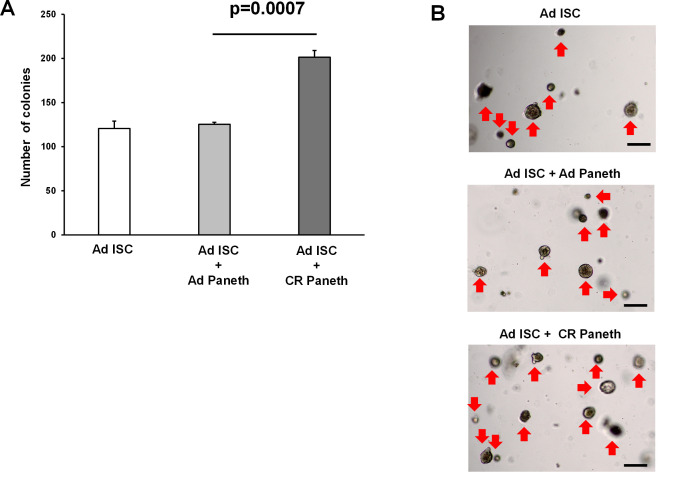
Organoid formation from the co-culture of isolated Paneth cells and intestinal stem cells (ISCs). ISCs and Paneth cells were isolated from ad libitum–fed (AD) and calorie-restricted (CR) mice by flow cytometry to more than 90% purity, and 2 × 10^3^ cells each were co-cultured. In trypan blue staining, the viability of isolated ISCs was 94.9%, whereas that of Paneth cells was 95.6%. The number of colonies was assessed at day 5 (3 wells/ group) (**A**), and representative images of the organoids (**B**) are shown. Original magnification: 40×. Scale bar: 100 μm. Values represent the mean ± SEM. Significant differences are denoted by p values (Student’s t-test). Red arrow marks organoids. Calorie restriction enhances the supportive function of the Paneth cell as described previously [5].

10. Quantify the number of colonies with lumen (Figure 4) under the microscope on day 5 of culture.

*Note: We recommend setting up more than two technical replicates to give a reliable count.*

## Validation of protocol

**Figure 5.**
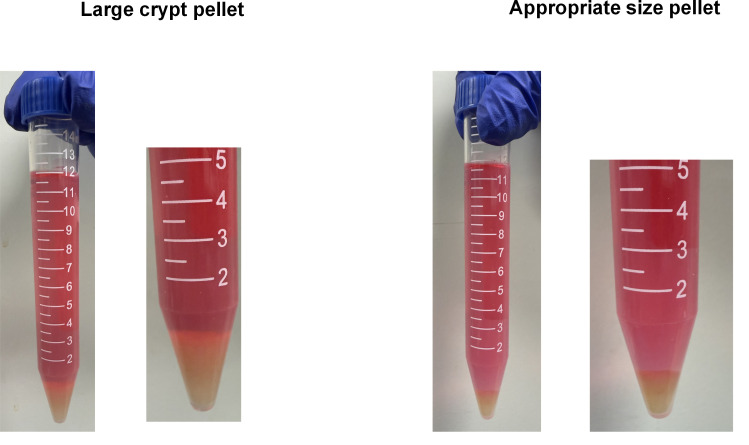
Example of an appropriate pellet size (corresponding to step B3, after centrifugation). Left: Cells in too large crypt pellets tend to be damaged in step B. Right: The example of appropriate size pellet in step B3. The pellet size should be adjusted to the size shown above.

This protocol has been used and validated in the following research articles:

- Isotani et al. [8]. Nicotine enhances the stemness and tumorigenicity in intestinal stem cells via Hippo-YAP/TAZ and Notch signal pathway. *eLife* (Figure 2).
- Igarashi et al. [7]. NAD+ supplementation rejuvenates aged gut adult stem cells. *Aging Cell* (Figure 2).
- Igarashi et al. [5]. mTORC1 and SIRT1 Cooperate to Foster Expansion of Gut Adult Stem Cells during Calorie Restriction. *Cell* (Figures 3–6).

## General notes and troubleshooting

**Troubleshooting**

Problem: Organoids do not grow, or the organoid size is too small.

Possible cause: ISCs and Paneth cells are damaged in the isolation procedures.

Solution: Replace TrypLE Express or the filter of Pipet-Aid. Make sure that steps B1–3 are performed without stopping. When the amount of crypt pellets is large, cells tend to be damaged in section B (Figure5). Then, adjust the size of the crypt pellets by discarding some of Fractions 2–5 in step A11.
